# The Functional Roles of Methionine and Arginine in Intestinal and Bone Health of Poultry: Review

**DOI:** 10.3390/ani13182949

**Published:** 2023-09-18

**Authors:** Guanchen Liu, Woo Kyun Kim

**Affiliations:** Department of Poultry Science, University of Georgia, Athens, GA 30602, USA; gl16770@uga.edu

**Keywords:** arginine, methionine, intestinal health, bone health, functional amino acids, poultry

## Abstract

**Simple Summary:**

Intestinal health and bone health are two major contributors to the well-being of poultry. Maintaining a balanced and healthy status of the intestines and bones can lead to optimal growth performance and productivity. Recent studies have illuminated the functional roles of certain amino acids, highlighting their unique contributions to various physiological processes and the synthesis of metabolically important molecules. Methionine and arginine are two notable examples of such amino acids. In this review, we explore how methionine and arginine may influence intestinal and bone health and the potential mechanisms behind these effects.

**Abstract:**

This review explores the roles of methionine and arginine in promoting the well-being of poultry, with a specific focus on their impacts on intestinal and bone health. The metabolic pathways of methionine and arginine are elucidated, highlighting their distinct routes within the avian system. Beyond their fundamental importance in protein synthesis, methionine and arginine also exert their functional roles through their antioxidant capacities, immunomodulating effects, and involvement in the synthesis of metabolically important molecules such as S-adenosylmethionine, nitric oxide, and polyamines. These multifaceted actions enable methionine and arginine to influence various aspects of intestinal health such as maintaining the integrity of the intestinal barrier, regulating immune responses, and even influencing the composition of the gut microbiota. Additionally, they could play a pivotal role in promoting bone development and regulating bone remodeling, ultimately fostering optimal bone health. In conclusion, this review provides a comprehensive understanding of the potential roles of methionine and arginine in intestinal and bone health in poultry, thereby contributing to advancing the nutrition, overall health, and productivity of poultry in a sustainable manner.

## 1. Introduction

The global poultry industry holds immense significance in meeting the continuously increasing demand for high-quality protein-rich food, driven by the continuous rise in the global population [1]. To achieve optimal growth and productivity of the birds, ensuring their health and well-being is essential. Among the critical factors that impact the performance of poultry, this review paper will specifically focus on two critical aspects: intestinal health and bone health.

The intestines are responsible for the digestion and absorption of essential nutrients from the feed consumed by poultry [2]. Furthermore, the intestinal tract is immensely important in the immune system of the animals. It serves as the physical barrier and also hosts abundant organized lymphoid tissue and immune effector cells, which collectively provide protection against pathogens and toxins [3,4,5]. Nevertheless, the intestines possess the most extensive exposed surface in the body [6]. The constant exposure to a wide range of potentially harmful substances makes them susceptible to many diseases, such as coccidiosis and necrotic enteritis, which have a significant impact on the poultry industry [7,8]. Maintaining a healthy intestinal tract has been a focus of research for decades, and this area is now gaining more attention due to the growing awareness of animal welfare, food safety, and the increasing public scrutiny towards the use of antibiotic growth promoters [9]. 

Likewise, bone health is also a significant contributor to the overall well-being of the birds [10,11]. The skeletal system serves as the foundation for the structure of the birds, providing support and enabling efficient movement [12]. Healthy bones are essential for preventing fractures, deformities, and other musculoskeletal issues, such as lameness, which can severely impact the comfort and performance of the birds [13]. Especially broilers undergo rapid growth, which places significant demands on their skeletal systems [12]. Ensuring proper bone development and strength is crucial to meet the birds’ physiological needs, prevent welfare issues, and optimize productivity.

In recent years, research has shed light on the functional roles of specific amino acids, with methionine (Met) and arginine (Arg) being two notable examples [14,15]. Met is an essential amino acid, and it is usually considered as the first limiting amino acid in corn and soybean meal-based diets for poultry [16]. Met plays a crucial role in protein synthesis as it serves not only as a fundamental building block of proteins but also as the initiating amino acid that is typically incorporated as the first residue to the polypeptide chain during translation [17]. Beyond its role in protein synthesis, it also contributes to the maintenance of oxidative balance due to its antioxidant capacity [18]. Met and its metabolite S-adenosylmethionine (SAM) act as methyl donors, a function that is essential for normal cellular metabolism [16]. Recent studies have further revealed its involvement in regulating immune responses [19,20]. Arg is also considered an essential amino acid for poultry. In addition to its significance in protein synthesis, it plays a vital role in various physiological processes. The immunomodulating effects of Arg have been widely reported because it is the precursor of nitric oxide (NO), which possesses potent immune regulatory effects and pathogen suppression capacity [15,21]. Arg has also been shown to affect lymphoid organ development and lymphocyte functions [22,23]. Furthermore, Arg contributes to the wound healing process by serving as the precursor of various polyamines and collagens [24,25]. Investigating the impacts of these two amino acids on poultry health holds the potential to devise effective strategies to enhance productivity, overcome challenges, and ensure better animal welfare. Abundant studies have been conducted to demonstrate the beneficial effects of Met and Arg supplementation on the intestinal health of poultry, whereas more research needs to be carried out to study its effects on the bone health of the birds (Table 1).

The objective of this review paper is to provide an overview of the current knowledge regarding the roles of Met and Arg in poultry intestinal health and bone health. We expect that the findings presented in this review will contribute to a deeper understanding of this subject and ultimately contribute to improvements in the health, performance, and productivity of poultry.

## 2. Metabolism of Methionine and Arginine

### 2.1. Methionine Metabolism

Methionine is an essential amino acid and usually considered the first limiting amino acid in corn and soybean meal based poultry diets [46]. It is classified as a sulfur-containing amino acid and is involved in a variety of important physiological processes, including protein synthesis, methylation reactions, and antioxidant activity [26,47].

**Figure 1:**
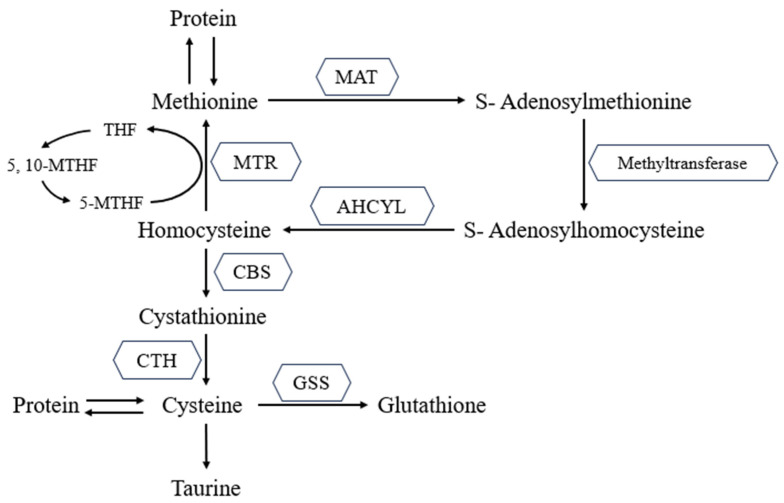
Methionine metabolism to glutathione [48,49]. THF, tetrahydrofolate; 5, 10-MTHF, 5, 10-Methylenetetrahydrofolate; 5-MTHF, 5-Methylenetetrahydrofolate; MAT, methionine adenosyltransferase; AHCYL, adenosylhomocysteinase like; MTR, methionine synthase; CBS, cystathionine-β-synthase; CTH, cystathionine gamma-lyase; GSS, glutathione synthase.

Methionine is metabolized through a complex series of enzymatic actions mainly in the liver (Figure 1). In a concise overview, the initial step of Met metabolism involves its transformation into SAM through a process known as methylation, catalyzed by the enzyme, methionine adenosyltransferase (MAT) [23,24]. SAM is a highly reactive molecule and serves as a key methyl donor for many important cellular processes, including the synthesis of DNA, RNA, proteins, and neurotransmitters. SAM is subsequently converted to S-adenosylhomocysteine (SAH), which is then further metabolized to homocysteine (Hcy). Hcy can then be remethylated back to Met with the active form of folate, 5-methyltetrahydrofolate (5-MTHF), being the methyl donor, thereby interlinking the metabolism of Met and folate. This process is catalyzed by methionine synthase (MTR) with vitamin B12 as the cofactor. Hcy alternatively undergoes transsulfuration to form cystathionine first and then cysteine. This process relies on vitamin B6 as a cofactor. Cysteine can then be further metabolized to form the vital endogenous antioxidant, glutathione (GSH) [48,49], or serve as a precursor for taurine synthesis [50].

### 2.2. Arginine Metabolism

Birds do not possess the entire urea cycle due to the absence of carbamoyl phosphate synthase (CPS1) and ornithine carbamoyltransferase (OTC). Consequently, they cannot synthesize Arg from metabolically generated ammonia or ornithine within the urea cycle [15,51]. Additionally, the limited activities of argininosuccinate synthetase (ASS) and argininosuccinate lyase (ASL) in birds hinder the adequate synthesis of Arg from citrulline [52]. As a result, the availability of Arg in birds is highly dependent on their diets, making Arg essential for poultry.

**Figure 2:**
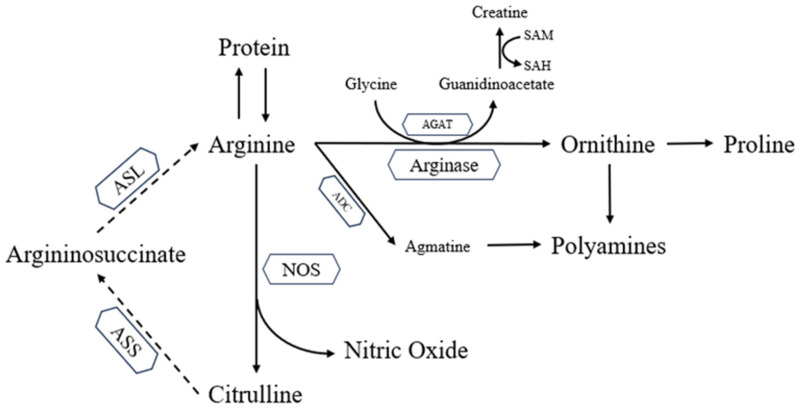
Arginine metabolism. Dotted lines represent pathways that are less active in birds than in mammals. ASL, argininosuccinate lyase; ASS, argininosuccinate synthetase; NOS, nitric oxide synthase; ADC, arginine decarboxylase; AGAT, arginine:glycine amidinotransferase.

In addition to its role in protein synthesis, Arg undergoes metabolism through four distinct pathways (Figure 2) [51,53]. One pathway involves a two-step enzymatic process where Arg, along with glycine, is metabolized to creatine by arginine:glycine amidinotransferase (AGAT), with SAM acting as the methyl donor. The other pathway involves the conversion of Arg to agmatine through the action of arginine decarboxylase (ADC). Agmatine is subsequently converted to the polyamine known as putrescine. Putrescine serves as a precursor for the synthesis of two other polyamines, namely, spermine and spermidine. 

However, the primary metabolic significance of Arg lies in two well-regulated pathways that compete for its utilization [51,54]. In the first pathway, Arg is metabolized by nitric oxide synthase (NOS) to produce NO. NO is a vital molecule involved in various metabolic processes, including immunity regulation and pathogen suppression. In the second pathway, Arg is metabolized by arginase to produce ornithine. Ornithine, in addition to being converted to putrescine, can also be transformed into proline [55]. Both polyamines and proline play crucial roles in cell proliferation, tissue repair, and wound healing processes.

## 3. Methionine and Arginine in Intestinal Health

### 3.1. Methionine and Arginine in Intestinal Development and Repair

The intestinal epithelium, which comprises a single layer of epithelial cells and a protein-rich mucus layer, has one of the highest turnover rates in the body [56]. The renewal of epithelial cells in the intestinal epithelium involves a highly coordinated process of cellular proliferation, differentiation, migration, and apoptosis. This constant cell turnover relies on ongoing protein synthesis, which is largely mediated through the activation of mTOR pathway [57]. In addition to serving as essential building blocks of proteins, Met and Arg have been shown to activate mTORC1 [58,59]. Met activates the mTOR signaling pathway by promoting the methylation of phosphatase 2A through the action of SAM. Furthermore, previous studies showed that SAM could bind to the S-adenosylmethionine sensor upstream of mTORC1 (SAMTOR), counteracting its inhibitory effects on mTORC1 activity [59,60]. On the other hand, Arg activates the mTOR pathway through inhibiting the activity of tuberous sclerosis complex 2, a protein that normally suppresses the mTOR pathway [61]. The mTOR pathway is essential for maintaining intestinal epithelial cell proliferation during both homeostasis and regeneration, as research showed that a disruption in this signaling pathway would lead to intestinal epithelial cell defects and hinder the intestinal regeneration [62,63]. 

The Wnt/β-catenin signaling pathway is also well established for its role in maintaining intestinal structure and homeostasis as it regulates the self-renewal and differentiation of the intestinal stem cells [64,65,66]. Interestingly, studies have also shown that both Met and Arg can activate the Wnt/β-catenin pathway to enhance the intestinal epithelial development [32,67]. Met is required for the sequestration of glycogen synthase kinase 3, which is an essential step in activating the Wnt signaling pathway [68]. The modulating effects of Arg on the Wnt/β-catenin signaling pathway can be attributed to the synthesis of NO, which is a known activator of this pathway [69]. Intriguingly, activation of the Wnt/β-catenin signaling pathway is closely regulated by the methylation of the Arg residues in Ras GTPase-activating protein-binding protein 1 (G3BP1) with SAM as the methyl donor. This linkage underscores the collaborative regulatory roles of Met and Arg in this pathway [68,70]. The activation roles of Met and Arg in both pathways contribute to the regenerative capacity and development of the intestine.

Beyond their roles in pathway activation, Met and Arg are indispensable for polyamine synthesis. Arg serves as the precursor for polyamine synthesis, while SAM, a product of Met metabolism, functions as the methyl donor in this process [71]. Polyamines are essential for intestinal epithelial renewal and repair, ensuring its important roles in cell proliferation, development, and migration [72,73]. Studies have demonstrated that providing dividing cells in the crypts with polyamines can stimulate mucosal growth and facilitate the repair of damaged mucosa [74,75,76]. The proposed mechanism underlying the stimulation effect of polyamines in mucosal growth involves their ability to regulate expression of various genes encoding growth promoting and inhibiting factors [71,77]. Polyamines are also shown to be vital for the expression of tight junction and adhesion junction proteins which maintain the intestinal barrier function [78,79]. 

Overall, Met and Arg are essential for the development and repair of the intestinal epithelium. They contribute to this process by activating pathways essential for intestinal regeneration and by participating in polyamine synthesis.

### 3.2. Antioxidant Effects of Methionine and Arginine on Intestinal Health

#### 3.2.1. Oxidative Stress

The integrity of the intestinal barrier can be disrupted by various factors, among which oxidative stress is a significant contributor. Reactive oxygen species (ROS) are byproducts generated during normal metabolic processes [80,81]. Under normal conditions, the production of ROS is balanced by the antioxidant system [82]. However, an excessive production of ROS or a decline in antioxidant defenses can disrupt this equilibrium, leading to the accumulation of ROS. The highly reactive ROS will react with the cellular components causing cellular damage, dysfunction, and apoptosis, which ultimately leads to impaired organ functions and the development of oxidative stress [80]. Several factors during poultry production can cause oxidative stress to the birds, such as nutritional factors like nutrient imbalances and feed toxins, environmental factors like heat stress and stocking density stress, as well as pathological factors [83,84,85]. 

Oxidative stress can damage the structure and function of tight junctions, resulting in compromised intestinal barrier functions and increased permeability [86]. Oxidative stress is also known to damage the intestinal epithelial cells directly, leading to further disruption of the barrier function and inflammation. The recruited macrophage and heterophils intensify the production of ROS, stimulating a positive feedback loop that exacerbates oxidative stress and inflammation [87,88]. Oxidative stress is also reported to cause morphometric changes in the intestinal tract by reducing the villi height and lowering the villus: crypt ratio, interfering with nutrient absorption [89,90].

#### 3.2.2. Antioxidant Effects of Methionine and Arginine

**Figure 3:**
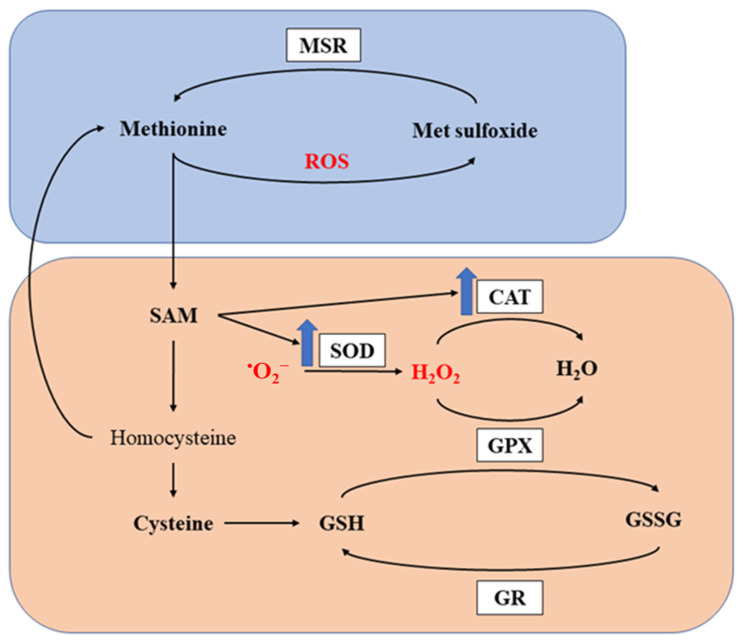
Antioxidant capacity of methionine. MSR, methionine sulfoxide reductase; SOD, superoxide dismutase; CAT, catalase; GPX, glutathione peroxidase; GR, glutathione reductase; ROS, reactive oxygen species; SAM, S-adenosylmethionine; GSH, glutathione; GSSG, glutathione disulfide. The orange and boxed represents the first and second mechanism for methionine to exert its antioxidant capacity. The blue arrows indicate upregulation of the enzymes.

Methionine is reported to exhibit potent antioxidant capacity through two major mechanisms [91,92]. Firstly, it produces antioxidant metabolites by undergoing metabolic processes as described in Figure 1. SAM, as one of the metabolites in the pathway, is not only an important methyl donor, but also a key metabolite modifying antioxidant enzymes, such as superoxide dismutase (SOD) and catalase (CAT) [91,92]. Such modification effects were confirmed by different research groups observing increased activities of antioxidant enzymes in birds fed Met supplemented diets [93,94,95,96,97]. Cysteine, another sulfur-containing amino acid, also exhibits antioxidant ability and is further metabolized to GSH, a well-known intrinsic antioxidant. Previous studies have also demonstrated increased GSH or improved GSH: glutathione disulfide (GSSG) ratio in broilers fed Met supplemented diets [84,98,99]. Secondly, Met residues in proteins can directly scavenge ROS. As a sulfur-containing amino acid, Met residues on the surface of proteins are readily oxidized. By scavenging ROS and being oxidized into methionine sulfoxide, Met can protect other critical components from oxidation, thus maintaining their integrity and function [17,70]. Furthermore, methionine sulfoxide can be reduced by methionine sulfoxide reductases (MSR) back to Met to restore its antioxidant capacity [20,100,101]. The antioxidant capacity of methionine and its metabolites is presented in Figure 3. 

Previous research has provided evidence on the beneficial effects of Met supplementation in intestinal health through its antioxidant capacity. Dietary Met supplementation in broilers improved the GSH:GSSG ratio and glutathione peroxidase activity as well as increased the villus height (VH) and ratio of villus height to crypt depth (VH:CD) with or without stocking density challenge [29]. Met supplementation also improved the tight junction protein expression, whereas it decreased the expression of proinflammatory cytokines in broilers under heat stress [30]. Another study also demonstrated that Met supplementation increased the concentration of GSH, while it reduced the malondialdehyde (MDA) content in the duodenum mucosa. The VH and VH:CD were again improved by Met supplementation [31]. Despite not being widely recognized for its antioxidant capacity, several studies have shown that Arg supplementation can improve the oxidative status and benefit intestinal health. In an in vitro study conducted in ovine intestinal epithelial cells [38], the researchers found that Arg supplementation significantly reduced the hydrogen peroxide-induced ROS production. Furthermore, Arg also increased levels of glutathione peroxidase and tight junction protein 1, whereas it decreased the level of proinflammatory cytokines. Moreover, supplementing Arg in broiler breeders improved the oxidative status of the breeder birds as well as the one-day-old offspring [37]. Another study conducted in rats challenged with sodium nitrite showed that Arg supplementation reduced the serum MDA content and increased GSH production [102]. Additionally, both Met and Arg have been shown to activate the nuclear factor erythroid 2-related factor 2–antioxidant responsive element (Nrf2-ARE) pathway, leading to the upregulation of antioxidant enzymes and the generation of antioxidant effects [103,104]. 

Considering the potent antioxidant capacity and effects of Met and Arg, incorporating both Met and Arg as functional dietary supplements for poultry holds significant promise in mitigating the detrimental effects of oxidative stress on intestinal health and the overall well-being of the birds. 

### 3.3. Methionine and Arginine in the Immune System

The intestinal tract is a major compartment of the immune system. The gut-associated lymphoid tissues (GALT) are estimated to comprise more immune cells than any other tissues [105]. The definable structures of GALT include lymphoid aggregates located within the lamina propria, Meckel’s diverticulum, Peyer’s patches, cecal tonsils, and bursa of Fabricius. In contrast to mammals, birds lack traditional lymph nodes; however, they possess numerous lymphoid aggregates that represent most of the secondary lymphoid tissues. Overall, the gut harbors diverse immune cell types, including heterophils, macrophages, dendritic cells, natural killer cells, and B and T cells, with the cellular compositions differing among different lymphoid tissues [106].

Methionine plays a critical role in both the humoral and cell-mediated immune responses in animals [107]. The impact of methionine on the humoral immune function of animals is primarily reflected in its effects on immunoglobulin levels in the body [108]. As for the cell-mediated immune responses, Met exerts regulatory effects on T cell activation and development [109]. Research showed that the activation of T cells is typically associated with an upregulation of Met transporters and SAM metabolism enzymes [110]. It was proposed that Met as well as its metabolites are taken up by the T helper cells for the synthesis of new protein and methylation of RNA and DNA, which drives activation, proliferation and differentiation of T cells [109,111]. Sufficient levels of methionine can significantly enhance antibody production as well as improve T cell proliferation in broilers [108,112,113,114]. Conversely, a deficiency of methionine significantly reduces the levels of immunoglobins in the bloodstream and inhibits the proliferation and differentiation of lymphocytes in broilers [115,116].

Numerous studies have extensively explored the immunoregulatory roles of Arg. Arg plays a significant modulatory role in immune function, primarily through the synthesis of NO by inducible NOS (iNOS) in macrophages [117,118]. NO is a versatile molecule that serves as a pivotal mediator in various immunological processes, including antimicrobial defense, immune cell regulation, and cytotoxicity [119,120]. Research has also shown its protective effects against protozoan infections [121,122]. Over the years, researchers have also shown that arginine availability is crucial for maintaining normal T cell proliferation and function. Rodriguez et al. [123] demonstrated that T cell function was significantly impaired under conditions of limited arginine supply, while this effect was completely reversed when arginine was replenished. It was proposed that arginine is indispensable for the expression of the T cell antigen receptor CD3ζ, which subsequently influences T cell function [25,123]. A recent study has also reported that Arg supplementation ameliorated the negative effect in *Eimeria*-infected broiler birds by enhancing the T cell function and elevating NO production [21].

Given the important roles of Met and Arg in immunity, the supplementation of Met and Arg could be a promising approach to enhance the immune response of the poultry intestine that constantly faces various challenges.

### 3.4. Methionine and Arginine in the Intestinal Microbiome

Numerous bacterial species inhabit the gastrointestinal tract (GIT) of chickens, with *Firmicutes*, *Bacteroidetes*, *Proteobacteria*, and *Actinobacteria* being the dominant ones at the phylum level [124]. Studies have revealed that maintaining a well-balanced microbiota profile is crucial for preserving intestinal health and normal functions [125]. For instance, these microbial communities break down polysaccharides to provide amino acids and short-chain fatty acids, serving as crucial energy sources for the epithelial cells [126]. Moreover, the healthy intestinal microorganism plays a protective role against pathogens by inhibiting the adherence and colonization of the pathogens as well as modulating the immune responses of the host [124,127].

The intestinal microbiota profile is known to be influenced by dietary factors [128]. Recent research has shed light on the impact of Arg supplementation on the intestinal microbiota of chickens. Multiple studies have consistently shown that Arg supplementation can lead to an increase in the relative abundance of *Firmicutes* and a decrease in *Proteobacteria* in the ileum [34,35,129]. At the genus level, Ruan et al. [35] demonstrated that Arg supplementation resulted in a higher relative abundance of *Romboutsia* and a lower abundance of *Clostridium sensu stricto 1*. Brugaletta et al. [130] found that in the ceca, Arg supplementation decreased alpha diversity and the relative abundance of *Proteobacteria*, while increasing the relative abundances of *Bacteroidetes* and *Lactobacillus salivarius*. There is limited evidence regarding the effects of Met supplementation on the chicken intestinal microbiota profiles. However, Kumar et al. [131] reported that Met supplementation could enhance glycolysis and energy generation by the cecal microbiota. Another study performed in germ-free pigs revealed that in the absence of intestinal microbiota, Met levels in the ileum and hindgut increased significantly, indicating that the intestinal microbiota were actively involved in Met metabolism [132]. 

In conclusion, the findings so far suggest that Arg supplementation has notable effects on the intestinal microbiota composition in chickens. However, further investigation is needed to explore the potential impacts of Met supplementation on the chicken intestinal microbiota.

## 4. Methionine and Arginine in Bone Health

### 4.1. Bone Formation, Growth and Remodeling

The majority of the skeleton is formed by the process called endochondral ossification involving the replacement of cartilage with bone tissue [133]. In the initial phase of this process, chondrocytes undergo proliferation, hypertrophy, and apoptosis, resulting in the formation of the cartilage extracellular matrix, primarily composed of type II and type X collagen and proteoglycans. Once the chondrocytes die, osteoblasts take over and secrete type I collagen and other non-collagenous bone matrix proteins such as osteopontin, osteonectin, and osteocalcin [134,135,136], depositing minerals to form the bone matrix and replace the cartilage. This replacement of cartilage initiates from the primary ossification center in the diaphysis. Concurrently, the same process occurs at both ends of the bone, known as the growth plate, leading to bone lengthening [137,138]. 

Bone remodeling is a continuous and essential process that takes place constantly. This process involves the coordinated activities of osteoblasts and osteoclasts [138]. Osteoblasts, which differentiate from mesenchymal stem cells (MSCs) under the regulation of runt-related transcription factor 2 (RUNX2), play a pivotal role in producing bone matrix proteins and driving bone formation [114]. On the other hand, osteoclasts, originating from hematopoietic stem cells upon activation by the receptor activator of NF-kB ligand (RANKL), function as bone-resorbing cells which break down the bone matrix, releasing calcium and phosphate back into the bloodstream [139]. Bone remodeling helps to repair microdamage in the bone matrix, thereby preventing the accumulation of old or damaged bone tissue and maintaining bone integrity. Additionally, bone remodeling aids in maintaining plasma calcium homeostasis [140]. The balance between osteoblast and osteoclast activities is tightly regulated by a variety of factors, including hormones, growth factors, mechanical stress, nutrients, and immune responses [138,141,142,143]. 

Recently, there has been a growing recognition of the significance of amino acids in skeletal metabolism [144,145]. Met and Arg, due to their vital roles in various metabolic processes including immune responses, antioxidant capacity and metabolically important compound synthesis, are important in maintaining bone development and normal functions [144].

### 4.2. Methionine and Arginine in Bone Metabolism

Previous studies have demonstrated that Met deficiency leads to deteriorated bone health. Ouattara et al. [42] conducted experiments on mice and rats subjected to a methionine-restricted diet. They observed a notable decline in key bone parameters, including volumetric bone mass density (BMD), bone mineral content, and bone microarchitecture parameters. Likewise, in another study conducted by Plummer et al. [43], the authors found that Met restriction in mice significantly decreased the cortical and trabecular BMD and also diminished bone volume and trabecular thickness. Remarkably, both studies reached a similar conclusion, suggesting that the reduction in bone health indicators may be linked to a decline in collagen synthesis and impaired osteoblast differentiation and functionality, which is further substantiated by the observed downregulation of RUNX2, the master regulator of osteogenesis. These observations were made in a mouse preosteoblast cell line cultured under low Met conditions and in the bone marrow of mice subjected to a Met deficient diet [42,43]. Fang et al. [146] also demonstrated that Met supplementation in fish could increase type 1 collagen synthesis. This finding further reinforces the significance of Met in collagen synthesis by osteoblasts. The mechanism behind this could be attributed to the important role Met plays in protein synthesis and mTOR signaling, as described in the previous section [144]. Vijayan [41] also demonstrated that Met supplementation to ovariectomized rats improved BMD and disrupted the development of osteoclasts. 

Arginine may contribute to bone development also by influencing collagen synthesis, because it is a precursor for proline and hydroxyproline, which are essential amino acids for collagen formation [55,144]. Furthermore, Van’T Hof and Ralston [147] stated in their review paper that NO is of significant importance in bone formation and reabsorption. It has been suggested that the regulatory effects of NO are primarily governed by endothelial nitric oxide synthase (eNOS), which is the predominant isoform of NOS in the bone [148]. In eNOS^(−/−)^ transgenic mice, researchers found significant abnormalities in bone formation [149,150]. Moreover, multiple studies have demonstrated that supplementation of NO donors can improve bone formation and strength in preclinical animal models [151] as well as reduce fracture risk in humans [152,153]. Given the importance of Arg in NO synthesis, Arg might further exert its influence in bone health. One recent study demonstrated that the absence of argininosuccinate lyase (ASL) in osteoblasts would lead to impairment of osteoblast differentiation [154]. Another study conducted in human MSCs further revealed that Arg supplementation in human MSCs enhanced osteoblastogenesis and inhibited adipogenesis through regulating the Wnt signaling pathway [45]. Previous studies have also shown that Arg supplementation in rats, broilers, and layers could improve bone mineralization and prevent bone loss [44,155,156]. 

Overall, through influencing collagen synthesis or NO metabolism, Met and Arg might exert their beneficial roles in bone development and formation. Because collagen is produced by mature osteoblasts, it is necessary to further investigate the roles of Met, Arg, and their metabolites on osteogenic differentiation of mesenchymal stem cells. 

### 4.3. Oxidative Stress, Intestinal Health and Bone Health

Oxidative stress is not only detrimental to intestinal health but also poses a great threat to bone health. Several reviews have extensively examined the influence of oxidative stress on bone health [157,158]. In essence, it triggers the apoptosis of osteoblasts and osteocytes, while promoting osteoclast formation through upregulating RANKL [159]. Consequently, this disruption generates an imbalance within the bone remodeling process, impeding mineralization and osteogenesis, and increasing bone resorption, ultimately leading to increased bone loss [11,157,160]. A recent study investigating the impact of hydrogen peroxide-induced oxidative stress on chicken compact bone-derived MSCs revealed that oxidative stress significantly increased apoptosis and decreased osteogenic differentiation of the cells, and this was accompanied by a universal decrease in osteogenesis-related gene expression and a decline in vitro mineralization [161]. Likewise, another study found that oxidative stress suppressed the expression of osteogenesis-related genes in chicken embryos, consequently impairing proper bone development [162]. Both studies confirmed the negative effects of oxidative stress on chicken bone development. Emerging observations have also indicated that oxidative stress is involved in various bone-related conditions including osteoporosis, bone tumor progression, and inflammatory joint diseases [163,164,165]. In vivo and in vitro studies have shown that antioxidant supplementations can mitigate oxidative stress and contribute to activation of osteoblast differentiation, mineralization, and reduction in osteoclast activity [166,167,168]. Considering the antioxidant capacities of Met and Arg discussed above, their supplementations might alleviate oxidative stress and benefit bone health.

Although appearing distinct, the intestinal health and bone health are intricately interconnected and the immune system emerges as a significant factor that bridges the gap between them [11]. On one hand, the bone marrow serves as the hemopoietic organ, offering a specialized environment for the development of hematopoietic stem cells, which are the shared origin of various immune cell types [169]. On the other hand, the intestine is considered the largest immune organ that is constantly encountering challenges [170]. As seen in multiple previous studies [171,172,173], the disturbance of the immune system caused by enteric diseases such as coccidiosis and enteric enteritis leads to the occurrence and development of bone disorders [11,169]. This process is once more propelled by the imbalance between osteoblast and osteoclast activities triggered by the upregulation of RANKL, which is induced by cytokines released from activated immune cells [174,175]. Acknowledging the protective role of Met and Arg in intestinal barrier functions and their immunomodulatory effects, it is plausible to speculate that these amino acids might contribute to the prevention of bone disorders by maintaining intestinal health.

### 4.4. Methionine and Arginine Effects on Bone Health in Different Growth Periods

Bone development in chickens differs during the early and late growth periods. During the early growth period (first 2–3 weeks of age), especially for the meat-type broilers, the cortex of the bones exhibits high porosity because the rate at which osteoblasts fill the osteon canals cannot match the rapid bone growth [176,177]. Consequently, birds are particularly vulnerable to developing skeletal abnormalities, emphasizing the significance of maintaining optimal bone health during this period [178]. As the birds age, the bones progressively mineralize as the osteoblasts mineralize and seal the inner canals [167]. Previous research showed that in broiler birds, the thickness and mineral density of tibia bones steadily increased with age, peaking at 4 to 5 weeks of age and remaining constant thereafter [179]. However, as the birds age, the increasingly higher body weight can place stress on the skeletal structure [180]. Furthermore, during the late growth period, the birds are more susceptible to oxidative stress due to the decline in their antioxidant defense system [181]. Additionally, they are more vulnerable to stress factors such as stocking density and high temperature [182]. These factors pose risks to bone health through different mechanisms compared with the challenges faced by the birds during the early growth period.

Given the distinct challenges to bone health during the early and late growth periods, it is reasonable to expect that the effects of dietary Met and Arg on bone development could vary. During the early growth phase, ensuring an adequate supply of Met and Arg may contribute to the production of more collagen and bone matrix proteins, supporting bone development. In contrast, during the late growth period, these functional amino acids might exert their effects on bone health through their antioxidant capacity and immunomodulating properties. However, it is worth noting that there is a limited body of research on this specific topic. Further studies are needed to better understand how Met and Arg supplementation can influence bone development during different growth stages and the mechanisms through which they exert their effects.

## 5. Potential Risks of Excessive Methionine and Arginine

Despite the considerable potential benefits associated with Met and Arg supplementation in poultry, excessive intake of these amino acids could give rise to unfavorable outcomes. One particular concern is the potential development of hyperhomocysteinemia due to excessive Met intake [183,184,185]. A high level of Hcy has been correlated with increased cardiovascular disease, inflammation, and compromised bone health [186,187,188]. Therefore, it is important to maintain normal blood Hcy levels when supplementing extra Met. One effective approach is to ensure adequate intake of B vitamins (B6, B12, and folate) to facilitate its conversion to methionine or cysteine [189] as they act as the cofactors for enzymes involved in its metabolism. However, folate and its derivatives are also known to be essential for the reproduction of certain pathogens such as *Eimeria* spp. [190,191]. Given the close relationship between Met and folate metabolism, excessive Met might favor the development of *Eimeria* spp. as one previous study showed that extra Met supplementation led to increased oocyst shedding in broilers [192]. Another noteworthy point is the antagonism between Arg and lysine when feeding excessive Arg to the birds, which may reduce food consumption, weight gain and Arg utilization in animal metabolism [193]. 

It is crucial to acknowledge that the functional roles of Met and Arg in various physiological processes, as discussed in earlier sections, could lead to an increased demand for these amino acids in stressful environments or during conditions of disease challenge. As previously suggested, the optimal Arg: Lys ratio increased in broilers under heat stress conditions [194]. Therefore, it is of utmost importance to supplement optimal amounts of Met and Arg in poultry diets, especially under challenging conditions, to produce the peak performance and productivity of the birds.

## 6. Conclusions

**Figure 4:**
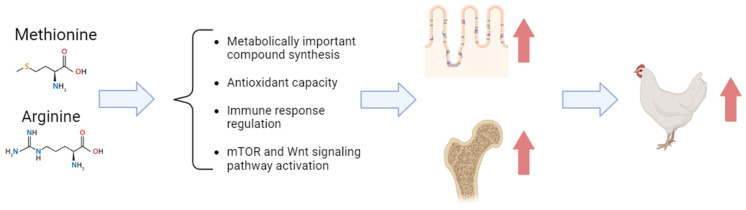
Schematic model illustrating potential mechanisms of methionine and arginine in enhancing intestinal and bone health in poultry. The red arrow in the figure indicates improvement.

In conclusion, this review comprehensively examines the functional roles of Met and Arg in promoting intestinal and bone health in poultry. These amino acids are integral not only to protein synthesis but also to diverse physiological functions. Within the intestinal context, Met and Arg contribute to development, repair, antioxidant defense, immune response, and microbiome modulation, collectively fostering optimal gut health. In terms of bone health, they are important in bone formation, growth, and remodeling, underscoring their significance in maintaining skeletal integrity (Figure 4). While their benefits are evident, caution is advised regarding excessive intake which could lead to adverse effects. Ultimately, a thorough understanding of the functions of Met and Arg offers insights into enhancing poultry nutrition and well-being. 

## Figures and Tables

**Table 1:** Summary of effects of dietary supplementation of methionine and arginine in intestinal and bone health in poultry from previous studies.

Methionine/Arginine Levels and Experiment Settings	Type of Birds/Models	Effects of Supplementation ^1^	Reference
Intestinal health:			
Methionine: DL-Met or L-Met at 60%, 80%, 100% of breeder recommendations. Settings: Mixed *Eimeria* spp. challenge	Male Cobb 500 broilers	Increased growth performance; reduced intestinal permeability; improved activities of antioxidant enzymes; and affected tight junction protein expression.	[26]
Methionine: 0.33%, 0.39%, 0.45%, 0.51%, or 0.57% of diet. Settings: Mixed *Eimeria* spp. challenge	Partridge Shank Broilers	Improved growth performance; increased relative weight of bursa of Fabricius; increased GPX activity, increased serum TAC; increased sIgA concentration; decreased lesion score.	[27]
Methionine: 0.24 or 0.45% of diet, supplemented with free Met or Met dipeptide. Setting: Mixed *Eimeria* spp. challenge.	Male Cobb 500 broilers	Increased TAC and decreased SOD activity in the jejunum; increased expression of B^0^AT1 and TLR5 in the jejunum.	[28]
Methionine: 0.35%, 0.4%, 0.45%, or 0.5% of diet. Setting: High stocking density	Male Arbor Acres broilers	Improved the GSH: GSSG ratio and GPX activity; increased the VH and VH:CD ratio.	[29]
Methionine: No Met supplementation or Met supplemented at recommended level. Setting: Heat stress.	Male Cobb 500 broilers	Improved growth performance; decreased the expressions of proinflammatory cytokines in jejunum and ileum; improved the tight junction protein expressions in the ileum.	[30]
Methionine: DL-Met or L-Met at 60%, 70%, 80%, 90% of breeder recommendations.	Mixed sex Ross 308 broilers	Increased the concentration of GSH and reduced MDA contents in duodenum mucosa; increased the VH and VH:CD ratio.	[31]
Methionine: 0.3% higher than the control diet supplemented with DL-Met or DL-methionyl-DL-Met.	White king breeding pigeons	Increased relative intestinal weight; increased VH and VH:CD ratio; increased expressions of cell proliferation markers, tight junction proteins, and PEPT1 in the jejunum; upregulated the Wnt/β-catenin signaling pathway.	[32]
Methionine: No Met supplementation or Met supplemented at recommended level.	Cobb broilers	Met deficiency decreased IgA^+^ B cell count; reduced contents of sIgA, IgA, IgG and IgM in duodenum and jejunum.	[33]
Arginine: 50% above recommendation in reduced protein diet. Setting: Mixed *Eimeria* spp. challenge.	Male Cobb 500 broilers	Improved growth performance; decreased intestinal permeability; increased macrophage NO production; increased bile IgA content; improved T cell functions.	[21]
Arginine: 0.3% higher than the recommendation. Setting: *Clostridium perfringens* challenge.	Male Arbor Acres broilers	Increased jejunal VH; balanced the ileal microbiota; increased relative abundance of KEGG pathways related to membrane transport, replication and repair, translation and nucleotide metabolism.	[34]
Arginine: 8.5, 9.7, 10.9, 12.1, and 13.3 g/kg of diet.	Female Qingyuan partridge chickens	Increased growth performance; increased activities of antioxidant enzymes; increased TAC in jejunum and ileum; decreased expression of proinflammatory cytokines in the ileum; increased sIgA production; improved ileal microbiota profile.	[35]
Arginine: 1.04, 1.14, 1.24, 1.34, 1.44% of diet. Setting: Mixed *Eimeria* spp. challenge.	Male Cobb 500 broilers	Improved growth performance; reduced intestinal permeability; increased expression of tight junction proteins.	[36]
Arginine: 0.96%, 1.16%, 1.36%, 1.56%, and 1.76% digestible arginine.	Female Arbor Acres broiler breeders	Increased TAC; increased activity of GPX, and decreased MDA in the breeder and the offspring.	[37]
Arginine: 350 μM in DMEM culture medium. Setting: Oxidative stress induced by hydrogen peroxide.	Ovine intestinal epithelial cells	Reduced hydrogen peroxide-induced ROS production; increased protein levels of GPX, tight junction protein 1, and nitric oxide synthase, whereas decreased the TNFα level.	[38]
Arginine: 11.1, 13.3 and 20.2 g/kg of diet. Setting: Mixed *Eimeria* spp. challenge.	Male Ross 708 broilers	Increased VH and decreased CD; increased goblet cell density; decreased expression of proinflammatory cytokine; increased mucosal maltase activity; 13.3 g/kg of Arg supplementation showed highest expression of anti-apoptosis gene and mTOR.	[39]
Arginine: 100, 105, and 110% of the recommendation. Setting: Mixed *Eimeria* spp. challenge.	Male Ross 308 broilers	Improved growth performance; increased VH and VH:CD ratio; decreased oocyst count.	[40]
Bone health:			
Methionine: 250 mg/kg body weight in drinking water.	Ovariectomized rats	Increased bone density; decreased development of osteoclasts by inhibiting the TLR-4/MyD88/NF-κB pathway.	[41]
Methionine: Study 1: 0.12% and 0.84% of mice diet. Study 2: α-MEM culture media with restricted sulfur amino acids.	Study 1: Male and female mice. Study 2: Mouse preosteoblast cell line.	Study 1: Met deficiency decreased bone mineral density, bone mineral content, and microarchitecture parameters; increased collagen degradation. Study 2: Met restriction delayed osteoblast differentiation and decreased expressions of genes regulating bone formation.	[42]
Methionine: 0.12% and 0.86% of diet.	Young male C57BL/6J mice	Decreased cortical bone density; decreased trabecular bone density, bone surface, trabecula and bone volume, and trabecular thickness; increased fragility; reduced expression of RUNX2 in bone marrow.	[43]
Arginine: 70, 80, 90, 100, and 110% of the recommendation.	Male Ross 308 broilers.	Improved growth performance, lean deposition, and bone mineral density.	[44]
Arginine: 0, 0.1, 1, and 10 µM in DMEM culture medium.	Human mesenchymal stem cells.	Increased osteogenic differentiation; increased expression of RUNX2, Dlx5, osterix, and wnt5a; decreased expression of adaptogenic transcription factors.	[45]

^1^ Abbreviations: Met, methionine; Arg, arginine; GPX, glutathione peroxidase; TAC, total antioxidant capacity; SOD, superoxide dismutase; GSH, glutathione; GSSG, glutathione disulfide; sIgA, secretory immunoglobin A; IgG, immunoglobin G; IgM, immunoglobin M; ROS, reactive oxygen species; MDA, malondialdehyde; NO, nitric oxide; TLR, toll like receptor; VH, villus height; CD, crypt depth; B^0^AT1, neutral amino acid transporter B(0)AT1; PEPT1, peptide transporter 1; mTOR, mammalian target of rapamycin; TNFα, tumor necrosis factor alpha; MyD88, Myeloid differentiation primary response 88; NF-κB, nuclear factor kappa light chain enhancer of activated B cells.
